# An integrated-molecular-beacon based multiple exponential strand displacement amplification strategy for ultrasensitive detection of DNA methyltransferase activity†
†Electronic supplementary information (ESI) available: Fig. S1 to S7 and Table S1. See DOI: 10.1039/c8sc05102j


**DOI:** 10.1039/c8sc05102j

**Published:** 2018-12-20

**Authors:** Yun-Xi Cui, Xue-Nan Feng, Ya-Xin Wang, Hui-Yu Pan, Hua Pan, De-Ming Kong

**Affiliations:** a State Key Laboratory of Medicinal Chemical Biology , Tianjin Key Laboratory of Biosensing and Molecular Recognition , Research Centre for Analytical Sciences , College of Chemistry , Nankai University , Tianjin 300071 , P. R. China . Email: kongdem@nankai.edu.cn ; Fax: +86-22-23502458; b Collaborative Innovation Center of Chemical Science and Engineering (Tianjin) , Tianjin , 300071 , P. R. China

## Abstract

DNA methylation is a significant epigenetic mechanism involving processes of transferring a methyl group onto cytosine or adenine. Such DNA modification catalyzed by methyltransferase (MTase) plays important roles in the modulation of gene expression and other cellular activities. Herein, we develop a simple and sensitive biosensing platform for the detection of DNA MTase activity by using only two oligonucleotides. The fluorophore labeled molecular beacon (MB) can be methylated by MTase and subsequently cleaved by endonuclease DpnI at the stem, giving a shortened MB. The shortened MB can then hybridize with a primer DNA, initiating a cycle of strand displacement amplification (SDA) reactions. The obtained SDA products can unfold new MB and initiate another cycle of SDA reaction. Therefore, continuous enlargement of SDA and exponential amplification of the fluorescence signal are achieved. Because the triple functions of substrate, template and probe are elegantly integrated in one oligonucleotide, only two oligonucleotides are necessary for multiple amplification cycles, which not only reduces the complexity of the system, but also overcomes the laborious and cumbersome operation that is always a challenge in conventional methods. This platform exhibits an extremely low limit of detection of 3.3 × 10^–6^ U mL^–1^, which is the lowest to our knowledge. The proposed MTase-sensing platform was also demonstrated to perform well in a real-time monitoring mode, which can achieve a further simplified and high-throughput detection. The sensing strategy might be extended to the activity detection of other enzymes, thus showing great application potential in bioanalysis and clinical diagnosis.

## Abstract 2

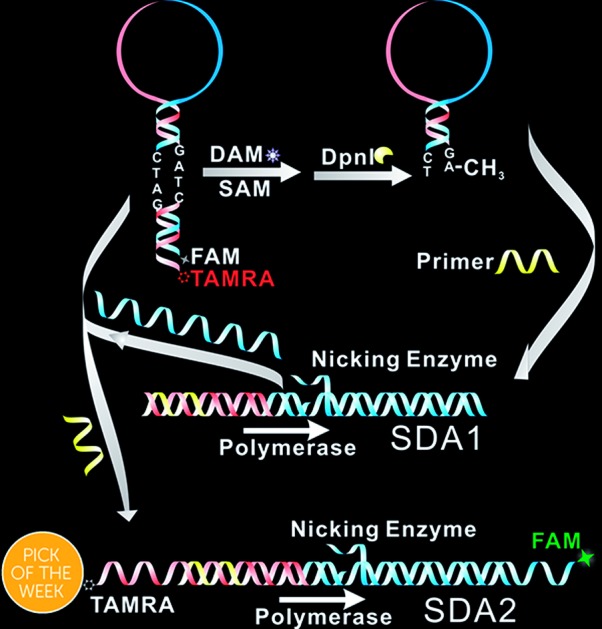
An ultra-sensitive biosensor using only two DNA oligos to initiate multiple signal amplification cycles.

## Introduction

Genomic DNA methylation is a crucial epigenetic DNA modification which commonly occurs in nature, and plays important roles in the regulation of gene expression.^1^,^2^ The DNA methylation process is catalyzed by a series of methyltransferases (MTase) that transfer a methyl group from *S*-adenyl methionine (SAM) to the C-5/N-4 positions of cytosine (C) or the N-6 position of adenine (A).^3^ To date, studies on cancer pathology have revealed that the abnormal pattern of DNA methylation induced by aberrant MTase activities is involved in various types of cancer, including breast, prostate, lung, liver and colon cancers.^4^–^9^ Therefore, the aberrant MTase activity can be recognized as a new generation of biomarkers for the early clinical diagnosis of cancer progression. Notably, inhibiting MTase to block DNA methylation may provide useful information for anticancer therapeutic applications.^10^–^12^ Hence, the development of a rapid and sensitive platform for the detection of MTase activity is of great significance in both pharmacological and biochemical research.

Conventional analytical methods for the detection of MTase activity include methylation-target polymerase chain reaction (PCR), radioactive labeling-based gel electrophoresis, and high-performance liquid chromatography (HPLC).^13^–^16^ However, most of these methods are time-consuming and harmful to biological systems because of their radioactive substances.^17^ Recently, various alternative approaches have been developed for an MTase assay, such as fluorescence, chemiluminescence, colorimetric and electrochemical methods.^18^–^28^ The emerging methods have attracted intense attention due to their cheap instrumentation and low toxicity. DNA-based biosensors, in particular, can provide high sensitivity and selectivity because of their programmable target-specific sequence and their computational controlled signal amplification strategy.^29^,^30^ A series of DNA-based biosensors have been applied in the analysis of MTase activity. For example, Yuan's group reported a fluorescence method based on exonuclease III (Exo III)-assisted isothermal cycling signal amplification;^31^ Zhang's group reported a surface-enhanced Raman scattering method based on strand displacement amplification (SDA) strategy;^32^ Zhu's group reported a chemiluminescence strategy combining hybridized chain reaction (HCR) and rolling circle amplification (RCA);^33^ and recently, Zhang's group reported an RNase HII-assisted single-ribonucleotide repair-mediated ligation-dependent cycling signal amplification method.^34^ Nevertheless, the complicated signal amplification process usually requires a sophisticated design of the DNA template and probe sequences.^34^ And numerous DNA oligos, which have to be involved in the system to assist the reaction, inevitably generate high background because of the nonspecific amplification.^33^ Thus, a biosensor with a simple design and high performance for the detection of MTase activity is still in urgent demand.

In this paper, by integrating the triple functions of substrate, template and reporter into one molecular beacon probe, we propose a novel strategy to design a succinct DNA-based biosensor *via* MTase-triggered multiple cycles of strand displacement amplification (SDA) reactions for exponential signal amplification. Such a biosensor can achieve ultrasensitive MTase activity detection in a simple “one-step” way, and the limit of detection is 3.3 × 10^–6^ U mL^–1^, which is the lowest we know of. Furthermore, the proposed sensing platform is demonstrated to perform well for the screening of MTase inhibitors, as well as the real-time monitoring of MTase activity.

## Materials and methods

### Chemicals and apparatus

All of the oligonucleotides used in this project (Table 1) were synthesized and purified by Sangon Biotech. Co. Ltd. (Shanghai, China). Dam and M. SssI methyltransferase (MTase), endonuclease DpnI, Klenow Fragment Polymerase (3′–5′ exo-) (KFP), nicking enzyme Nb.BbvcI, *S*-adenyl methionine (SAM), EcoRI enzyme and the corresponding buffer solution were obtained from New England Biolabs (Beijing, China). Deoxyribonucleoside triphosphate (dNTP) was purchased from Tiangen Biotech. Co. Ltd. (Beijing, China). All other chemicals were applied in analytical grade and were ordered from Solarbio (Beijing, China). All solutions for the reaction were prepared with ultrapure water which was purified by a Milli-Q water purification system (>18.25 MΩ cm^–1^).

**Table 1:** The DNA sequence applied in the MTase assay process

Application	Name	Sequence
Dam MTase detection	Molecular beacon	TTCGGATCTTCCCGCCCTACCCATTTTTTTTTTCCTCAGCTTTTGGAAGATCCGAA
	Primer 0	TTTTTTCTTCC
	Primer 1	TTTTTTCTTCCA
	Primer 2	TTTTTTCTTCCAA
EcoRI detection	Molecular beacon	TGCGAATTCCCGCCCTACCCATTTTTTTTTTCCTCAGCTTTTTCGGGAATTCGCA
	Primer 0	ATATATACCCG
	Primer 1	ATATATACCCGA
	Primer 2	ATATATACCCGAA

Fluorescence spectra were measured by a Hitachi RF-5301 fluorescence spectrometer (Hitachi Ltd., Japan). The values of the melting temperature (*T*_m_) of the DNA oligos were obtained from the Mfold website (University at Albany-State University of New York, USA). The gel electrophoresis results were obtained by a Gel Documentation System (Huifuxingye, Beijing, China). A real-time quantification PCR (RT-qPCR) assay was performed in a commercial StepOnePlus™ Real-Time PCR instrument (Applied Biosystems, USA).

### MTase assisted strand displacement amplification

The molecular beacon template was first diluted in 1× Dam MTase reaction buffer (50 mM Tris–HCl, 10 mM EDTA, 5 mM 2-mercaptoethanol, p H7.5) to a final concentration of 2 μM; then 160 μM SAM, and different concentrations of Dam MTase were added to the reaction system to a total volume of 25 μL. The mixture was reacted at 25 °C for 2 h. Next, 10× CutSmart buffer (500 mM potassium acetate, 200 mM Tris-acetate, 100 mM magnesium acetate, 1 mg mL^–1^ BSA, pH 7.9) and 10 U of DpnI were added for the cleavage reaction and the total volume was adjusted to 50 μL. The cleavage reaction was performed at 37 °C for 1 h, followed by heat deactivation at 80 °C for 20 min. Finally, 10 μL of the truncated molecular beacon (1 μM), 10 μL of primer DNA (1 μM), 10 μL of 10× CutSmart buffer, 10 μL of 10 mM dNTP, 0.5 U of KFP and 1 U of Nb.BbvcI were mixed together to a total volume of 100 μL and incubated at 37 °C for 1 h followed by 80 °C heat deactivation for 20 min as the SDA process.

### Fluorescence measurement

After the SDA process, the fluorescence signal of the product was directly measured by the fluorescence spectrometer. The excitation wavelength was set at 490 nm, and the emission spectrum from 500 nm to 650 nm was collected for further analysis.

### Gel electrophoresis assay

The SDA product was analyzed through polyacrylamide gel electrophoresis (PAGE). After the reaction, 15 μL of the product was mixed with 3 μL of prepared-loading buffer. The mixture was loaded into a 10% polyacrylamide gel contained in 1× TBE buffer (9 mM Tris base, 9 mM boric acid, 0.2 mM EDTA, pH 7.5). The PAGE was performed under 120 V constant voltage at room temperature for 50 min. The gel was stained with ethidium bromide. The stained gel was visualized using the Gel Documentation Imaging System.

### Inhibition of Dam MTase activity

10 μL of the molecular beacon substrate (10 μM) was first mixed with different concentrations of gentamycin or 5-fluorouracil, and pre-incubated in 1× Dam MTase reaction buffer at 37 °C for 30 min. Then 10 U mL^–1^ Dam MTase and 160 mM SAM were added into the reaction mixture and incubated at 37 °C for 2 h. Next, 10× CutSmart buffer and 10 U of DpnI were added into the mixture and another incubation was performed at 37 °C for 1 h, followed by heat deactivation at 80 °C for 20 min. Finally, the SDA reaction was performed at 37 °C for 1 h followed by 80 °C heat deactivation for 20 min. The fluorescence signal was measured as described above, and the relative activity (RA) of the Dam MTase was calculated based on eqn (1):

1 RA = (*F*_G_ – *F*_0_)/(*F*_R_ – *F*_0_)

where *F*_G_, *F*_R_, and *F*_0_ represent the fluorescence intensity in the presence of different concentrations of gentamycin, in the absence of gentamycin and in the absence of MTase, respectively.

### Detection of Dam MTase activity in a real sample

A total volume of 100 μL of a sample containing 10% human serum spiked with various concentrations of Dam MTase was prepared for the Dam MTase activity assay. The procedure for the fluorescent measurement was the same as that described above.

## Result and discussion

### Design of the MTase biosensor

**Scheme 1:**
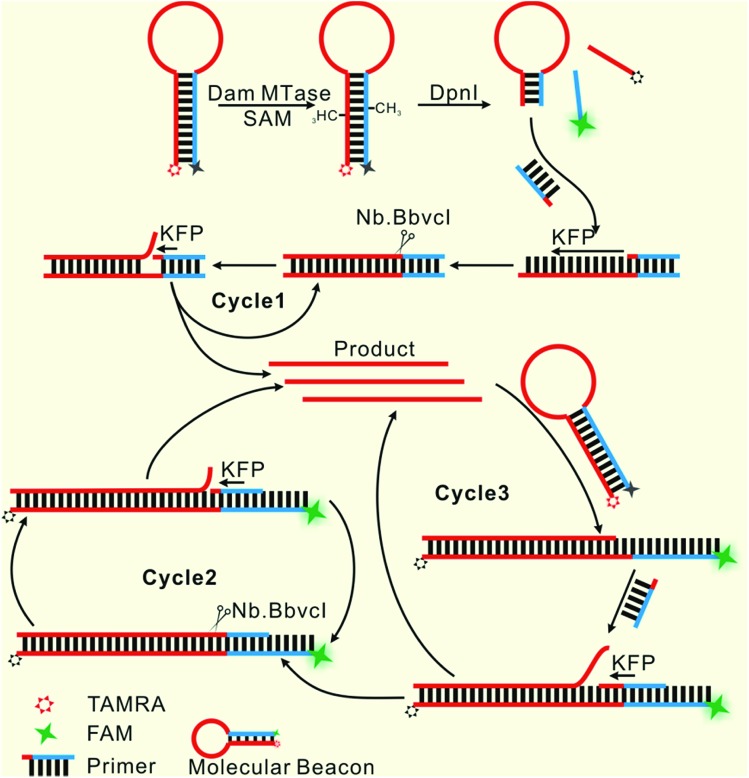
Illustration of the probe-reporter integrated biosensor platform mechanism.

Herein, only two oligonucleotides are used. One is a hairpin-like molecular beacon (MB) oligonucleotide (molecular beacon, Table 1) which plays the triple roles of substrate for MTase recognition, template for MTase-triggered strand displacement amplification (SDA) and probe for amplified signal output. The other is a single-stranded linear oligonucleotide used as an SDA primer (primer, Table 1). The two ends of the MB are labeled with the fluorophores FAM and TAMRA, respectively, and the FAM fluorescence is efficiently quenched by TAMRA due to close contact. The double-stranded stem of MB contains a specific 5′-GATC-3'/3′-CTAG-5′ region where the adenines (A) will be methylated in the presence of Dam MTase and subsequently be cleaved by endonuclease DpnI at the methylated positions. The cleavage reaction leads to two consequences: the separation of FAM from TAMRA to give recovered fluorescence and the hybridization of the primer with the shortened MB. The hybridization would not affect the intact molecular beacon because the longer hairpin structure is much more stable (*T*_m_ = 69.1 °C) compared to the shortened hairpin (*T*_m_ = 42.7 °C). Such a hybridization will initiate the first cycle of SDA reaction under the catalysis of Klenow Fragment Polymerase (KFP) and nicking enzyme Nb.BbvcI to give amplified SDA products. These products will in turn recognize and open the original MB substrates that were not previously methylated by Dam MTase. As a result, the fluorescence of FAM, which was quenched by the TAMRA quencher, is recovered due to the separation between the two ends of the MB. In addition, primers will hybridize on these unfolded MBs to initiate the second cycle of SDA, resulting in the release of product strands, which could be repeatedly used to unfold new MBs. More importantly, this cycle of SDA could also provide new products. Collectively, since the product strands used to trigger new SDA reactions can be provided by three routes—products of the first cycle of SDA, products of the second cycle of SDA and the repeatedly used products—SDA reactions will be continuously enlarged and exponential signal amplification can be achieved (Scheme 1). Integrating three functions into one MB substrate will not only simplify the design of the sensing system, but also avoid the high background signal induced by the nonspecific amplification because of the reduction in the number of DNA oligos included in the complicated signal amplification process reported before. In the low MTase concentration range, most of the MB substrates might not be cleaved, but these parts can also participate in signal amplification *via* the next cycle of SDA. Therefore, the issues of low utilization of probes and/or templates, which are often suffered by most DNA-based biosensors, are completely overcome.

### Feasibility of the designed biosensor

**Fig. 1:**
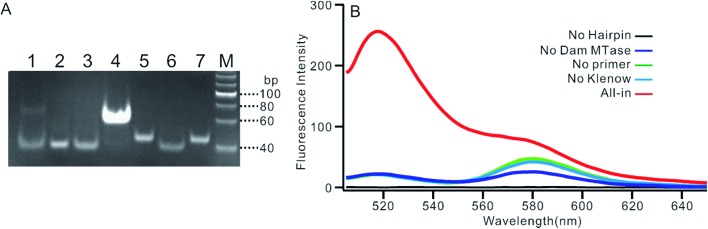
(A) PAGE result of the proposed sensing platform. Line M: DNA marker, line 1: SDA proceeding on shortened MB without Nb.BbvcI, line 2: SDA proceeding on shortened MB with primer 0, line 3: SDA proceeding on shortened MB without primers, line 4: complete SDA proceeding on shortened MB only, line 5: DpnI treated MB without Dam MTase, line 6: shortened MB cut by DpnI, line 7: MB only. (B) Fluorescent spectrum in response to the addition of different components.

We employed polyacrylamide gel electrophoresis (PAGE) to monitor the reaction process of the proposed sensing platform. As shown in Fig. 1A, after the treatment with Dam MTase and DpnI to the MB, a band with a faster migration rate appeared, according to the cleavage of the template (Fig. 1A, line 6). In contrast, the MB treated with DpnI showed no significant difference from the original MB (lines 5 & 7), indicating that the MTase was necessary in the MB-cleavage process. Moreover, the expected SDA products were only observed when the shortened MB was employed. As shown in Fig. 1A, lines 1–4, a new band with a slower migration rate was observed, indicating that the amplification proceeded and an unfolded signal reporter had been generated. However, the band of the product did not appear if we applied the original MB for the SDA (line 3), showing that the MTase-associated DNA cleavage was necessary for the initiation of the SDA process. In addition, compared to the one without nicking enzyme Nb.BbvcI, the band of the product was much brighter after the enzyme was added in (lines 1 & 4), showing that the SDA process would be completed only in the presence of both Klenow and Nb.BbvcI.

We also verified the mechanism of the proposed sensing platform by a fluorescent assay. As shown in Fig. 1B, a significant increase in the fluorescence intensity could be observed only in the presence of all of the materials. No obvious signal could be observed in the absence of any of the enzymes or DNA oligos, indicating that the fluorescent signal was obtained from the SDA process particularly initiated by the target enzyme. In addition, the value of the fluorescence intensity (*I*_t_) at 520 nm is about 6 times higher in the presence of nicking enzyme Nb.BbvcI (Fig. S1†), and the value of Δ*I*_t_ (subtracting the background from the signal intensity) is about 25 times higher, compared with the one without Nb.BbvcI. This result indicated that the nicking enzyme-mediated amplification cycles could efficiently increase the sensitivity of the sensing platform.

### Optimization of the experimental conditions

**Fig. 2:**
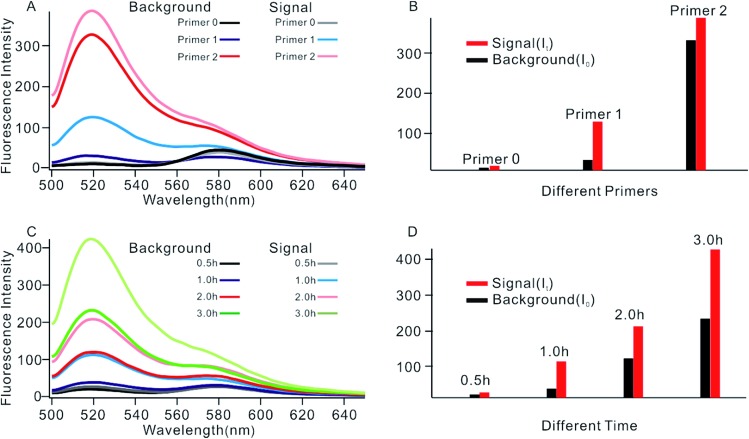
(A) Fluorescent spectrum in response to different primers. (B) Measurement of the fluorescence intensity in response to different primers. (C) Fluorescent spectrum in response to different reaction times. (D) Measurement of the fluorescence intensity in response to different reaction times.

In order to obtain the best sensing performance, biosensor design and some important experimental conditions were optimized. First of all, the best primer was selected (Fig. 2). An ideal primer should efficiently hybridize and unfold the shortened MB but have no effect on intact MB. We noticed that when a short primer (*e.g.*Table 1, primer 0 with 11 nucleotides) was used, a very low fluorescence signal was given even in the presence of 0.1 U mL^–1^ Dam MTase, indicating that an exponential SDA reaction was not initiated because this primer was too short to hybridize with the shortened MB. When the primer length increased to 13 nucleotides, however, high background was given even in the absence of Dam MTase. The reason was that the primer was too long; it can hybridize and unfold intact MB, resulting in the occurrence of undesirable amplification reactions. By comparing several primers with different lengths, primer 1 of 12 nt length provided an acceptable background and the best signal-to-noise ratio (*I*_t_/*I*_0_, where *I*_t_ and *I*_0_ represent fluorescence intensity with and without Dam MTase). Thus primer 1 was applied in subsequent investigations.

The reaction time of the SDA reaction was also critical because increasing reaction time will certainly give an enhanced signal intensity but might also increase the possibility of undesirable side reactions, thus inducing high background. By synchronously monitoring the time-dependent fluorescence changes of the sensing platform with and without Dam MTase, 1 h was selected as the optimal reaction time due to its acceptable background and the highest *I*_t_/*I*_0_ value. Similarly, using *I*_t_/*I*_0_ as a criterion, the concentration of primer 1 was optimized at 100 nM (Fig. S2†), and the amounts of KFP and Nb.BbvcI were selected at 0.5 and 1 units in a total volume of 100 μL, according to previous research.^35^

### Sensitivity and selectivity of the biosensor

**Fig. 3:**
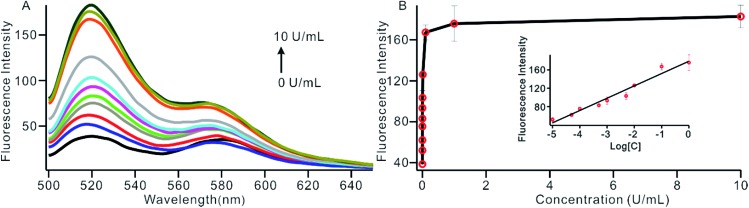
(A) The fluorescent spectrum in response to different concentrations of Dam MTase, from low to high; the curve revealed MTase concentrations of 0, 1 × 10^–5^, 5 × 10^–5^, 1 × 10^–4^, 5 × 10^–4^, 1 × 10^–3^, 5 × 10^–3^, 0.01, 0.1, 1 and 10 U mL^–1^, respectively. (B) Variance of the fluorescence intensity with the concentration of Dam MTase in the range of 1 × 10^–5^ to 1 U mL^–1^. The insert shows the linear relationship between the fluorescence intensity and the logarithm of the concentration of Dam MTase.

To investigate the sensitivity of the biosensor, we measured the fluorescence intensity at various concentrations of Dam MTase under the optimal conditions (Fig. 3A). The fluorescence intensity was enhanced as a function of MTase concentration from 10^–5^ to 10 U mL^–1^ (Fig. 3B). In logarithmic scales, the fluorescence intensity exhibited a linear correlation with the concentration of Dam MTase over a wide range from 10^–5^ to 1 U mL^–1^ (Fig. 3B). The regression equation is *I*_t_ = 178.38 + 26.79 × log *C*_MTase_ with a correlation coefficient of 0.973, where *I*_t_ and *C*_MTase_ represent the fluorescence intensity and the Dam MTase concentration, respectively. The limit of detection (LOD) was estimated to be 3.3 × 10^–6^ U mL^–1^ based on 3 times the standard deviation over the blank response (3*σ*/*S*). Notably, the LOD of this biosensor is the lowest to our knowledge. The sensitivity of this biosensor platform has improved by as much as 2 orders of magnitude compared with that of rolling circle amplification (RCA) based chemiluminescence assay,^21^ by 3 orders of magnitude compared with that of the methylation response DNAzyme colorimetric assay,^22^ by 3 orders of magnitude compared with that of quantum dots-mediated FRET assay,^36^ and as well as being an improvement over other methods.^37^–^39^ The extremely low LOD could be attributed to (i) the high amplification efficiency because of making full use of the integrated probe-reporter MB substrates in the SDA amplification process; (ii) multiple highly efficient SDA cycles were included to enhance the signal; and (iii) low background due to few oligos being applied in the whole system.

**Fig. 4:**
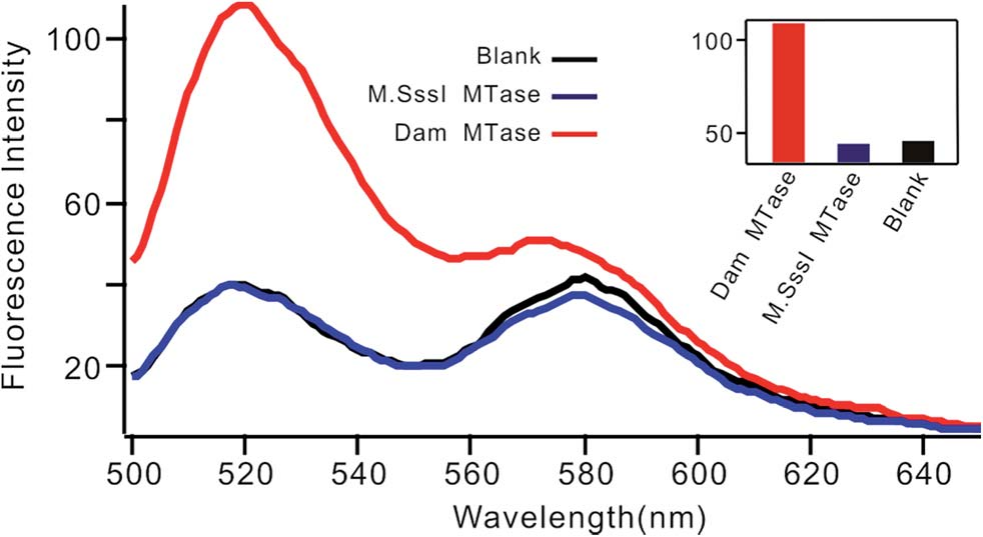
Fluorescent spectrum in response to 0.01 U mL^–1^ Dam MTase and 0.1 U mL^–1^ M.SssI MTase. The insert shows the fluorescence intensity at 520 nm according to the spectrum.

Selectivity is an important characteristic of biosensors. To evaluate the selectivity of this biosensor, we introduced M.SssI MTase as a potential interference enzyme. M.SssI MTase is also a methyltransferase; it can methylate the cytosine within a sequence of 5′-C-G-3'/5′-C-G-3′ in double-stranded DNA.^20^ As shown in Fig. 4, the fluorescence intensity increased significantly in the presence of Dam MTase. In contrast, no distinct signal change was observed in the presence of M.SssI MTase compared to the blank control even when the concentration of M.SssI MTase was 10 times higher than that of Dam MTase, thus revealing the high selectivity of the proposed biosensor toward Dam MTase.

### MTase inhibitor screening

**Fig. 5:**
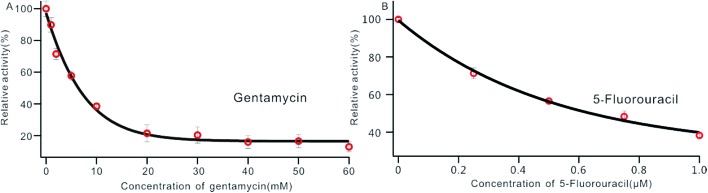
Variance of the relative activity of Dam MTase in response to different concentrations of gentamycin (A) and 5-fluorouracil (B). The concentration of Dam MTase is 10 U mL^–1^.

DNA MTase was reported as a significant biomarker as well as an important diagnostic target in recent research.^36^ Pharmacologically inhibiting DNA MTase activity may alter the DNA methylation levels in cells, which is concerned in a variety of cancers.^39^ Thus, the screening of inhibitors for MTase has attracted intense interest. To evaluate the potential of our biosensor for DNA MTase inhibition assay, we applied gentamycin, which is widely used as an inhibitor of methyl transferase,^40^ as a model inhibitor. Collecting the fluorescence intensity at 520 nm with different concentrations of gentamycin added into the reaction system, we defined the relative activity of the Dam MTase based on eqn (1). As shown in Fig. 5A, the relative activity of Dam MTase reduced gradually with increasing concentration of gentamycin from 0 to 60 mM. The half maximal inhibition (IC_50_) is defined as the concentration of the inhibitor applied to achieve a 50% relative activity. According to the calibration curve in Fig. 5A, the IC_50_ value of gentamycin is calculated to be 8.02 mM. Here, the value is consistent with the value (10.0 mM) obtained by an Exo III-mediated fluorescence based assay.^31^ Similarly, the inhibition function of 5-fluorouracil was also investigated using the proposed platform. According to Fig. 5B, the IC_50_ value of 5-fluorouracil is calculated to be 0.71 μM. The value obtained by our sensing platform approximates those reported in recent research.^18^,^37^ This result demonstrates that this biosensor can be used for the inhibitory capacity evaluation of DNA MTase inhibitors and thus for inhibitor screening, holding great potential in pharmacological applications.

### MTase activity assay in real sample

We also investigated the capability of the proposed biosensor in the application of a Dam MTase assay in a human serum sample. Different concentrations of Dam MTase were spiked into a 10% serum sample and the recoveries were calculated by comparing the measured Dam MTase activity and the amount of Dam MTase added into the system (see Fig. S3† for the spectrum result). As shown in Table 2, the recoveries were found to be 94.0% to 111.0% according to the measurement, with relative standard deviations (RSD) ranging from 3.4% to 9.0%. The result revealed that the new biosensor was reliable for the detection of MTase in a real sample.

**Table 2:** Recovery studies of Dam MTase in human serum samples

Sample	Added (U mL^–1^)	Measured (U mL^–1^)	Recovery	RSD
1	0.05	0.047	94.0%	8.1%
2	0.1	0.11	110.0%	9.0%
3	0.5	0.47	94.0%	6.4%
4	1	1.11	111.0%	3.4%

### MTase activity assay in real-time SDA mode

**Fig. 6:**
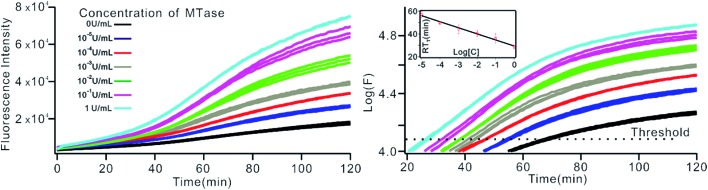
The Dam MTase activity assay by RT-qPCR. (A) Fluorescence–time plot and (B) logarithm of fluorescence–time plot obtained by RT-qPCR system under different concentrations of Dam MTase. The insert shows the linear relationship between the RT_t_ value and the logarithm of the concentration of MTase at a threshold of 4.1.

The above MTase activity assay was conducted in an end-point detection mode, in which the fluorescence intensity was detected after the SDA reactions. To further simplify the experimental operations, the feasibility of MTase activity quantification in a real-time mode was investigated. In such a mode, the fluorescence change of the sensing system was instantaneously recorded during the SDA reaction *via* a commercial, real-time quantitative PCR instrument (StepOnePlus™ Real-Time PCR system, ABI, United States). As shown in Fig. 6A, plots of fluorescence (*F*) *vs.* reaction time (*T*) were obtained with different MTase concentrations. Similar to the conventional real-time PCR, a unique signal-processing method can be applied. That is, log(*F*)–*T* plots were constructed on the basis of the obtained *F*–*T* plots (Fig. 6B), and the RT_t_ values, the reaction time at which the log(*F*) reaches a set threshold, were calculated. On a logarithmic scale, a linear relationship (*R*^2^ = 0.985) was obtained between the RT_t_ value and the concentration of MTase, in the range of 1 × 10^–5^ to 1 U mL^–1^. The linear regression equation was calculated as RT_t_ = 29.26 – 5.33 × log[*C*_MTase_]. From Fig. 6, it was found that the log(*F*)–*T* plots of the systems containing 1 × 10^–5^ U mL^–1^ Dam MTase could be easily discriminated from those of the blank control, thus confirming the excellent detection sensitivity of the real-time detection mode. Compared to the end-point mode, the real-time mode has simplified operations due to the elimination of a post-amplification signal detection procedure, thus endowing the method with increased high-throughput detection capability. In addition, since the signal amplification and detection are synchronously performed, the reaction tubes can be directly discarded after SDA reactions without opening the lids, thus greatly reducing the risks of amplification product carryover contamination, a big challenge that exponential nucleic acid amplification reactions have to face.

### Generality of the proposed sensing strategy

By a slight modification of the sequence of the stem region of the MB, the proposed MTase-sensing strategy might be easily extended to the design of other sensors targeting different enzymes. For example, the design of a restriction endonuclease sensor can be achieved by simply replacing the MTase recognition sequence on MB with a restriction endonuclease recognition one. As a proof-of-concept, a sensing platform for EcoRI, a restriction endonuclease that can specifically recognize and cleave 5′-GAATTC-3′/3′-CTTAAG-5′, was designed. Similar to the proposed Dam MTase-targeted sensing platform, the hairpin structure was less stable after cleavage by EcoRI (*T*_m_ decreased from 73.3 °C to 42.0 °C), and thus could be unfolded by a short primer, initiating the SDA cycles. We confirmed the feasibility of the designed platform by gel electrophoresis. (see Fig. S4† for the details). The sensor was then demonstrated to work well for the highly sensitive detection of enzyme activity with good selectivity as well (Fig. S5 and S6†). This result indicated that the proposed sensor design strategy might be used for designing a series of biosensors for the activity analysis of a broad spectrum of biologically important enzymes.

## Conclusion

In summary, a novel exponential signal amplification strategy was developed and successfully used for biosensor design. By highly integrating the three roles of substrate, template and reporter into one oligonucleotide, the biosensor platform can be constructed by using only two oligonucleotides, thus greatly reducing the complexity of the sensing system and simplifying the sensing operations. The potential background generated by the numerous DNA oligonucleotides involved in the multiple amplification steps can also be avoided. The unique exponential signal amplification mode ensures the full and effective utilization of the oligonucleotides in sensing systems, thus endowing the biosensor with an extraordinarily high sensitivity. The constructed biosensor was demonstrated to perform well for the ultrasensitive detection of Dam MTase activity, as well as the screening of the potential inhibitor of Dam MTase, and the quantificational analysis of Dam MTase in a real sample. The LOD of Dam MTase was as low as 3.3 × 10^–6^ U mL^–1^, which is the lowest so far, compared with reported methods (Table S1†). In addition, by simply modifying the enzyme recognition sequence in the oligonucleotide, the sensor design strategy was demonstrated to be easily extendable to the detection of other enzymes (*e.g.* restriction endonuclease EcoRI), thus showing great potential in pharmacological assay and in clinical diagnosis.

## Conflicts of interest

The authors declare no competing financial interest.

## Supplementary Material

Supplementary information
