# Supplementary material for: An integrated-molecular-beacon based multiple exponential strand displacement amplification strategy for ultrasensitive detection of DNA methyltransferase activity
Source: Chem Sci. 2018 Dec 20;10(8):2290–7. doi: 10.1039/c8sc05102j (PMC6385671; doi:10.1039/c8sc05102j)
Supplement: Supplementary file 1 [file SC-010-C8SC05102J-s001.pdf]

Electronic Supplementary Material (ESI) for Chemical Science.
This journal is © The Royal Society of Chemistry 2018

An integrated-molecular-beacon based multiple exponential strand displacement amplification strategy for ultrasensitive detection of DNA methyltransferase activity

Yun-Xi Cui[a,b], Xue-Nan Feng[a,b], Ya-Xin Wang[a,b], Hui-Yu Pan[a], Hua Pan[a], De-Ming Kong[a,b,*]

[a] State Key Laboratory of Medicinal Chemical Biology, Tianjin Key Laboratory of Biosensing and Molecular Recognition, Research Centre for Analytical Sciences, College of Chemistry, Nankai University, Tianjin 300071, P R China

[b] Collaborative Innovation Center of Chemical Science and Engineering (Tianjin), Tianjin, 300071, P R China.

Materials and Methods

Chemicals and apparatus

All of the oligonucleotides used in this project were synthesized and purified by Sangon Biotech. Co. Ltd. (Shanghai, China). Dam and M. SssI methyltransferase (MTase), endonuclease DpnI, Klenow Fragment Polymerase (3'-5' exo-) (KFP), nicking enzyme Nb.BbvcI, *S*-adenyl methionine (SAM), EcoRI enzyme and the corresponding buffer solution were obtained from New England Biolabs (Beijing, China). Deoxyribonucleoside triphosphate (dNTP) was purchased from Tiangen Biotech. Co. Ltd. (Beijing, China). All other chemicals applied in analytical grade were ordered from Solarbio (Beijing, China). All solutions for the reaction were prepared with ultrapure water which was purified by a Milli-Q water purification system(>18.25 MΩ/cm).

Fluorescence spectra was measured by Hitachi RF-5301 fluorescence spectrometer (Hitachi. Ltd., Japan). Gel electrophoresis result was obtained by a Gel Documentation System (Huifuxingye, Beijing, China). Real-time quantitation PCR (RT-qPCR) assay was performed in a commercial StepOnePlus™ Real-Time PCR instrument (Applied Biosystems, USA).

MTase assisted strand displacement amplification

The molecular beacon template was first diluted in 1 × Dam MTase reaction buffer (50mM Tris-HCl, 10mM EDTA, 5mM 2-mercaptoethanol, pH7.5) to a final concentration of 2 μM, then 160 μM SAM, and different concentration of Dam MTase was added in to the reaction system as a total volume of 25 μL. The mixture was reacted at 25 °C for 2h. Next, 10 × CutSmart buffer (500 mM potassium acetate, 200 mM tris-acetate, 100 mM magnesium acetate, 1 mg/mL BSA, pH 7.9) and 10 U of DpnI were added in for the cleavage reaction and the total volume was adjusted to 50 μL. The cleavage reaction was performed under 37 ℃ for 1h and followed with heat deactivation at 80℃ for 20 min. Finally, 10 μL of the truncated molecular beacon (1μM), 10 μL of primer DNA (1μM), 10 μL of 10 × CutSmart Buffer, 10 μL of 10mM dNTP, 0.5U KFP and 1U Nb.BbvcI were mixed together in a total volume of 100 μL and incubated in 37 ℃ for 1 h followed with 80℃ heat deactivation for 20 min as the SDA process.

Fluorescence measurement

After the SDA process, the fluorescence signal of the product was directly measured by the fluorescence spectrometer. The excitation wavelength was set as 490 nm, and the emission spectrum from 500 nm to 650 nm was collected for further analysis.

Gel electrophoresis assay

The SDA product was analyzed through polyacrylamide gel electrophoresis (PAGE). After reaction, 15 μL of the product was mixed with 3 μL prepared-loading buffer. The mixture was loaded into a 10% polyacrylamide gel contained in 1 × TBE buffer (9 mM Tris base, 9 mM boric acid, 0.2 mM EDTA, pH 7.5). The PAGE was performed under 120 V constant voltage at room temperature for 50 min. The gel will be stained with ethidium bromide. The stained gel was visualized using Gel Documentation Imaging System.

Inhibition of Dam MTase activity

10 μL of the molecular beacon substrate (10 μM) was first mixed with different concentrations of gentamycin and pre-incubated in 1 × Dam MTase reaction buffer at 37 °C for 30 min. Then 10 U/mL

Dam MTase, 160 mM SAM were added into the reaction mixture and incubated at 37 ℃ for 2 h. Next, 10 × CutSmart buffer and 10 U of Dpn I were added into the mixture and another incubation was performed at 37 ℃ for 1 h, followed with heat deactivation at 80℃for 20 min. Finally, SDA reaction was performed at 37 ℃ for 1 h followed with 80℃ heat deactivation for 20 min. The fluorescence signal was measured as described above, and the relative activity (RA) of the Dam MTase was calculated based on eqn (1):

$$RA = (F_G - F_0)/(F_R - F_0) \ldots\ldots \quad (1)$$

where F_G, F_R, and F_0 represent the fluorescence intensity in the presence of different concentration of gentamycin, in the absence of gentamycin and in the absence of MTase, respectively.

Detection of Dam MTase activity in real sample

A total volume of 100 μL of sample containing 10% human serum spiked with various concentrations of Dam MTase was prepared for the Dam MTase activity assay. The procedure of the fluorescent measurement was same as that described above.

EcoRI assisted strand displacement amplification

The molecular beacon template was first diluted in 1 × CutSmart buffer (50 mM potassium acetate, 20 mM tris-acetate, 10 mM magnesium acetate, 0.1 mg/mL BSA, pH 7.9) to a final concentration of 2 μM, then different concentration of EcoRI was added in to the reaction system as a total volume of 25 μL. The mixture was reacted at 37 ℃ for 1h and followed with heat deactivation at 80℃ for 20 min. Finally, 10 μL of the truncated molecular beacon (1μM), 10 μL of primer DNA (1μM), 10 μL of 10 × CutSmart Buffer, 10 μL of 10mM dNTP, 0.5U KFP and 1U Nb.BbvcI were mixed together in a total volume of 100 μL and incubated in 37 ℃ for 1 h followed with 80℃ heat deactivation for 20 min as the SDA process.

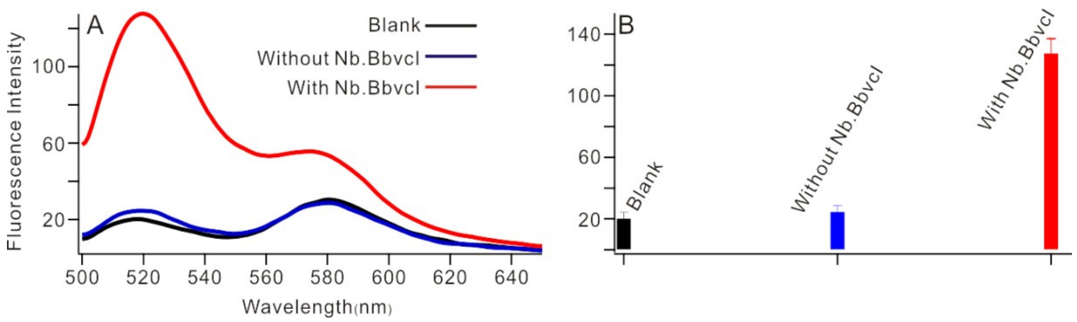

Figure S1: (A) The fluorescent spectrum in response to reaction with and without the nicking enzyme Nb.BbvcI, in presence of 0.01 U/mL of Dam MTase (B) Variance of the fluorescence intensity with and without the nicking enzyme Nb.BbvcI

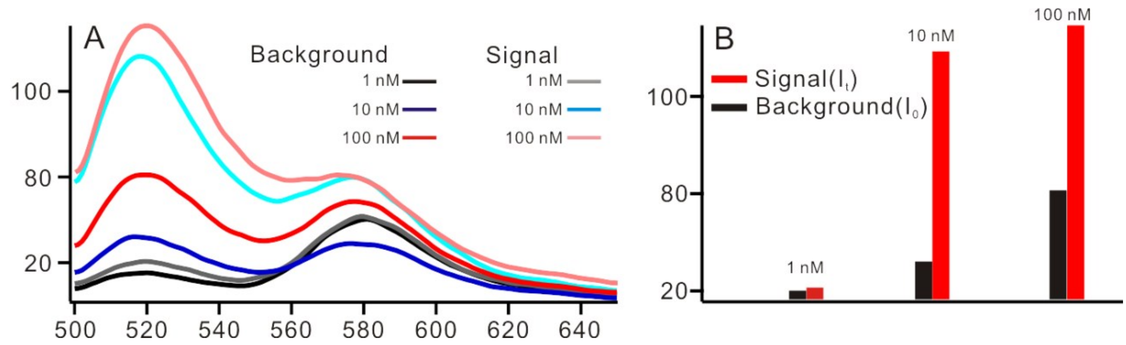

Figure S2: (A) Fluorescent spectrum in response to different concentration of primers (B) Measurement of the fluorescence intensity in response to different concentration of primers

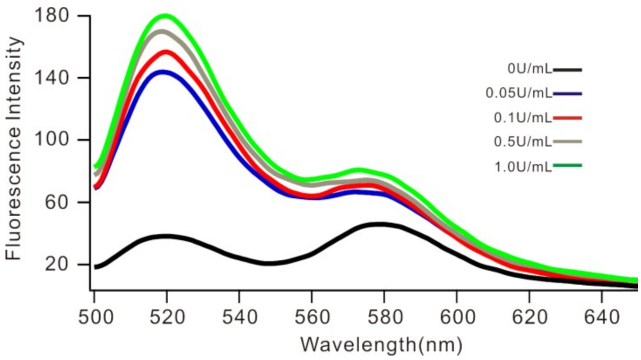

Figure S3: The fluorescence spectrum obtained under different concentration of Dam MTase in 10% serum sample.

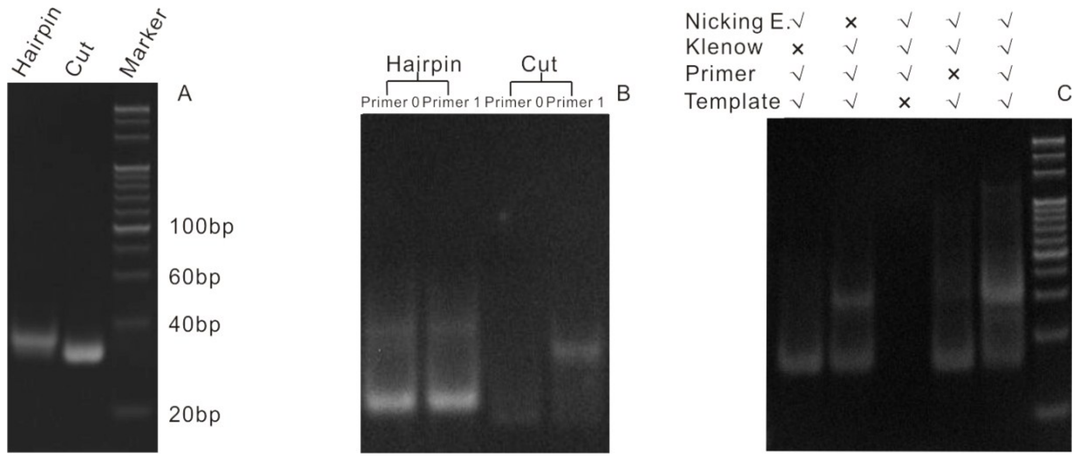

Figure S4: The polyacrylamide gel electrophoresis assay of the EcoRI sensing platform. (A) The hairpin template (See Table 1 for the sequence) with and without EcoRI cleavage (B) The amplification of the template with and without EcoRI cleavage, using primer with different length (C) The amplification of the template with different component addition

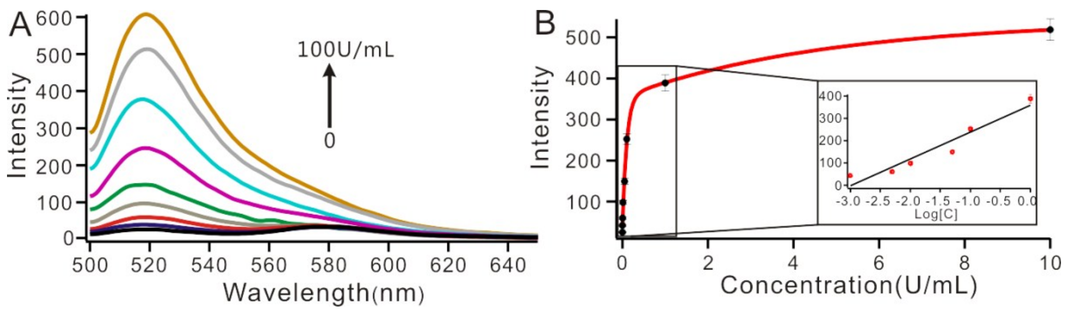

Figure S5: (A) The fluorescence spectrum obtained under different concentration of EcoRI, from low to high, the concentration of the EcoRI is 0, 0.001, 0.005, 0.01, 0.05, 0.1, 1, 10 and 100U/mL, respectively (B) The relationship between the fluorescence intensity at ~520 nm and the concentration of the EcoRI, insert indicate the linear fitting of the intensity to the logarithm of the concentration with a range of 0.001U/mL to 1 U/mL. The regression equation is $I_t = 358.21 + 120.63\times\log_{10}[C_{EcoRI}]$ with a correlation coefficient of 0.925

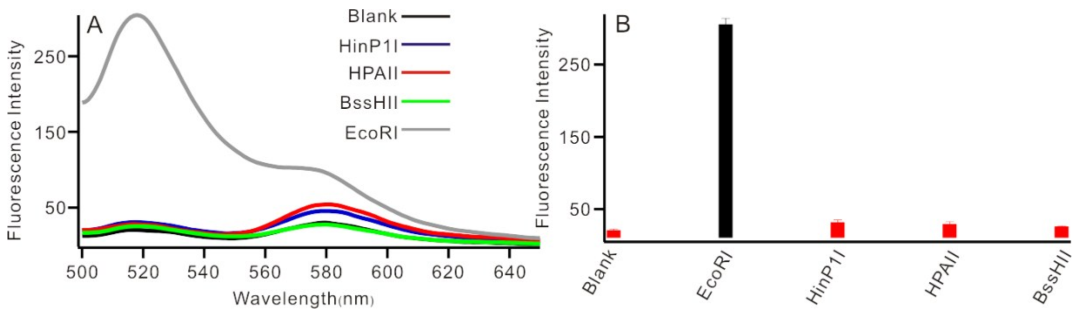

Figure S6: (A) The fluorescent spectrum in response to the EcoRI sensing platform with different kinds of endonuclease (B) Measurement of the fluorescence intensity for the EcoRI sensing platform with different kinds of endonuclease.

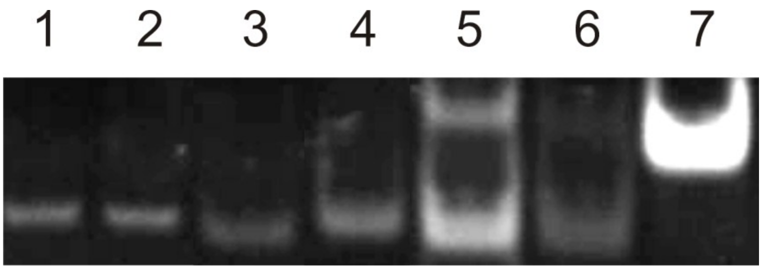

Figure S7: (A) PAGE result of the proposed sensing platform. line 1:MB only, line 2: DpnI treated MB without Dam MTase, line 3: Methylated MB cut by DpnI, line 4: SDA proceed on intact MB only, line 5: SDA proceed on shortened MB without Nb.BbvcI, line 6: SDA proceed on shortened MB with Primer0, line 7: complete SDA proceed on shortened MB.

Method	Strategy	Detection of limit(U/ml)	Reference
Colorimetry	DNA modified meso-$SiO_2@Fe_3O_4$	0.73	[1][1]
Colorimetry	GQ-based DNAzyme	0.25	[2][2]
SERS	Target triggering primer generation	$2.57x10^{-4}$	[3][3]
Electrochemistry	Exo III assisted	0.031	[4][4]
Electrochemistry	DNA SDR recycling	0.03	[5][5]
Electrochemistry	DNA-AuNP network	0.02	[6][6]
Fluorescence	RNase HII assisted	$4.8x10^{-6}$	[7][7]
Fluorescence	HCR with DNAzyme recycling	$7.23x10^{-4}$	[8][8]
Fluorescence	SDA and ERCA integrated	$8.1x10^{-5}$	[9][9]
Our method	SDA based on integrated probe-reporter	$3.3x10^{-6}$	

Table S1: Detection performance comparison of the proposed biosensor with other methods

References:

1. P. Liu, K. X. Zhang, R. R. Zhang, H. S. Yin, Y. L. Zhou and S. Y. Ai, *Anal. Chim. Acta*, 2016, **920**, 80-85.
2. W. Li, Z. L. Liu, H. Lin, Z. Nie and J. H. Chen, *Anal. Chem.*, 2010, **82**, 1935-1941.
3. Y. Li, C. F. Yu, H. X. Han, C. S. Zhao and X. R. Zhang, *Biosens. Bioelectron.*, 2016, **81**, 111-116.
4. X. Li, Z. P. Xie, W. Wang, Y. L. Zhou, H. S. Yin, Z. Q. Yang and S. Y. Ai, *Anal. Methods*, 2016, **8**, 2771-2777.
5. J. W. Zhou, X. H. Zhang, E. H. Xiong, P. Yu, X. Y. Li and J. H. Chen, *J. Electroanal. Chem.*, 2016, **781**, 304-309.
6. L. Hong, J. Wan, X. J. Zhang and G. F. Wang, *Talanta*, 2016, **152**, 228-235.
7. L. J. Wang, X. Han, C. C. Li and C. Y. Zhang, *Chem. Sci.*, 2018, **9**, 6053-6061.
8. B. Y. Jiang, Y. L. Wei, J. Q. Xu, R. Yuan and Y. Xiang, *Anal. Chim. Acta*, 2017, **949**, 83-88.
9. W. L. Cui, L. Wang, X. W. Xu, Y. Wang and W. Jiang, *Sens. Actuator B-Chem.*, 2017, **244**, 599-605.
